# Transcranial Magnetic Stimulation Efficacy and Future Directions in Major Depressive Disorder: A Comprehensive Review

**DOI:** 10.1192/j.eurpsy.2026.11441

**Published:** 2026-07-31

**Authors:** J. Ashton, D. Hyland

**Affiliations:** Psychiatry, University of Liverpool, Liverpool, United Kingdom

## Abstract

**Introduction:**

Major Depressive Disorder (MDD) constitutes a major global health burden, with a substantial proportion of patients showing inadequate response to pharmacotherapy and psychotherapy. Transcranial Magnetic Stimulation (TMS) has emerged as a safe and effective neuromodulatory intervention, particularly for treatment-resistant depression (TRD). However, differences in efficacy, treatment sustainability, and patient-specific outcomes across TMS protocols remain under investigation.

**Objectives:**

This review aimed to evaluate the comparative efficacy of repetitive TMS (rTMS) and accelerated TMS (aTMS) in TRD, assess treatment durability, explore age-related response patterns, and examine the impact of TMS in combination with pharmacological or psychotherapeutic interventions.

**Methods:**

A comprehensive literature review was conducted, including peer-reviewed studies published up to March 2025. Databases were systematically searched for randomised controlled trials, meta-analyses, and observational studies evaluating clinical outcomes, response durability, and demographic predictors of treatment efficacy.

**Results:**

Both rTMS and aTMS demonstrated significant antidepressant effects in TRD populations. aTMS achieved comparable response and remission rates to rTMS while reducing overall treatment duration. Sustained benefits were observed for up to six months post-treatment; however, relapse rates increased thereafter, with response persistence declining to approximately 46.3% at 12 months. Findings regarding age-related responsiveness were inconsistent, suggesting that older adults may exhibit variable outcomes depending on stimulation parameters and comorbidities. Combined TMS approaches with antidepressant medication or psychotherapy produced superior clinical improvement compared to monotherapy, indicating a synergistic interaction.

**Image 1:**
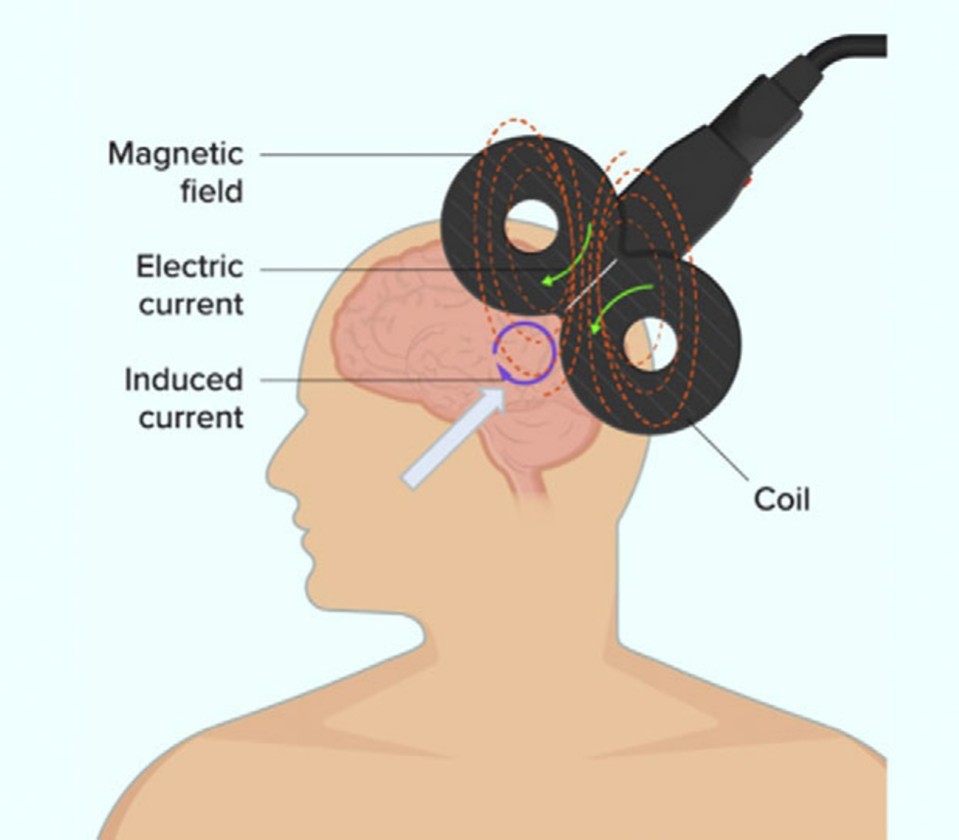

**Conclusions:**

TMS represents an effective and well-tolerated intervention for TRD, with accelerated protocols offering time-efficient alternatives. Despite robust short-term efficacy, maintenance strategies are required to prolong therapeutic gains. Future research should prioritise personalised TMS protocols guided by neurobiological and demographic markers to optimise response prediction and long-term outcomes across heterogeneous patient populations.

**Reference:**

Jaekl et al. Knowable Magazine 2023; Harvard Teaching Hospital, Beth Israel Deaconess Medical Centre

**Disclosure of Interest:**

None Declared

